# Validated prediction of pro-invasive growth factors using a transcriptome-wide invasion signature derived from a complex 3D invasion assay

**DOI:** 10.1038/srep12673

**Published:** 2015-08-05

**Authors:** Bettina Oehrle, Gerald Burgstaller, Martin Irmler, Stefan Dehmel, Jessica Grün, Tiffany Hwang, Susanne Krauss-Etschmann, Johannes Beckers, Silke Meiners, Oliver Eickelberg

**Affiliations:** 1 Comprehensive Pneumology Center, University Hospital of the Ludwig-Maximilians-University Munich and Helmholtz Zentrum München, Member of the German Center for Lung Research, 81377 Munich, Germany; 2 Institute of Experimental Genetics, Helmholtz Zentrum München, German Research Center for Environmental Health (GmbH), 85764 Neuherberg, Germany; 3 Technical University Munich, Chair of Experimental Genetics, 85350 Freising-Weihenstephan, Germany

## Abstract

The invasion of activated fibroblasts represents a key pathomechanism in fibrotic diseases, carcinogenesis and metastasis. Invading fibroblasts contribute to fibrotic extracellular matrix (ECM) formation and the initiation, progression, or resistance of cancer. To construct transcriptome-wide signatures of fibroblast invasion, we used a multiplex phenotypic 3D invasion assay using lung fibroblasts. Microarray-based gene expression profiles of invading and non-invading fibroblasts demonstrated that 1,049 genes were differentially regulated (>1.5-fold). Unbiased pathway analysis (Ingenuity) identified significant enrichment for the functional clusters ‘invasion of cells’, ‘idiopathic pulmonary fibrosis’, and ‘metastasis’. Matrix metalloprotease 13 (MMP13), transforming growth factor (TGF)-β1, Caveolin (Cav) 1, Phosphatase and Tensin Homolog (Pten), and secreted frizzled-related protein (Sfrp) 1 were among the highest regulated genes, confirmed by qRT-PCR and Western Blotting. We next performed *in silico* analysis (Ingenuity Pathway Analysis) to predict mediators that induced fibroblast invasion. Of these, TGFβ1, epidermal growth factor (EGF), fibroblast growth factor (FGF) 2, and platelet-derived growth factor (PDGF)-BB were tested in our 3D invasion assay and found to significantly induce invasion, thus validating the transcriptome profile. Accordingly, our transcriptomic invasion signature describes the invading fibroblast phenotype in unprecedented detail and provides a tool for future functional studies of cell invasion and therapeutic modulation thereof using complex phenotypic assays.

Fibroblast invasion through interstitial tissue is one substantial step in the dynamic process of tissue remodelling and repair, a process that also plays a pivotal role in fibrogenesis^1^, carcinogenesis, and metastasis^2^,^3^. Cell migration through the three-dimensional (3D) architecture of most tissues, in particular the lung, requires an invading phenotype that enables fibroblasts to penetrate the extracellular matrix (ECM) and cross the basal membrane. This migratory program is an inherent feature of many cells, such as fibroblasts or leukocytes, yet it can also be specifically increased during inflammation or tissue wounding^4^. Importantly, the mode of interstitial migration differs between various cell types: leukocytes are mainly found to display an integrin-independent amoeboid mode of migration, while most cancer cells and fibroblasts display mesenchymal movements^5^.

In tissue fibrosis, such as idiopathic pulmonary fibrosis (IPF), areas of activated fibroblasts, are believed to represent the site of increased production, secretion, and deposition of ECM^6^,^7^. In the lung, α-smooth muscle actin (αSMA)-positive fibroblasts originate from the sub-pleural region and form a fibroblastic reticular structure into the interstitium^8^. It is widely assumed that this process requires an invading fibroblast phenotype^9^. During injury, repair, and fibrosis, a plethora of soluble mediators are released by different cell types, which activate and attract resident fibroblasts and/or induce transformation of epithelial or pleural mesothelial cells (PMC) into mesenchymal-like cells^10^,^11^,^12^,^13^. Such aberrantly activated mesenchymal cells exhibit an exceptional plasticity with respect to proliferation, apoptosis, ECM deposition, activation, and tissue invasion^11^. In agreement with this concept, an increased collagen-invading capacity of human lung fibroblasts derived from IPF patients has recently been reported in 2D assays^14^. Furthermore, keloid fibroblasts are suggested to exhibit a highly invasive character, inducing infiltration of these fibroblasts into surrounding healthy skin tissue^15^. Enhanced fibroblast invasion, however, does play a decisive role in carcinogenesis and metastasis, as the tumour microenvironment is increasingly recognized as a major feature of tumour progression^3^. Here, the crosstalk between cancer cells and cells of the neoplastic stroma are important factors for invasive tumour growth and metastasis^16^,^17^. In particular, cancer associated fibroblasts (CAF) have been reported to represent key determinants in cancer progression^2^. Studies using squamous cell carcinoma (SCC) cells and stromal fibroblasts revealed that force- and protease-mediated matrix remodelling by the fibroblast is required for collective invasion of the SCC cells^18^, indicating that invading fibroblasts are able to direct carcinoma cells through stromal tissue.

In the current study, we hypothesized that pathway analysis of the transcriptome-wide signature of invading lung fibroblasts by comparative microarray analyses, using a collagen-based invasion assay previously established in our lab^19^, will identify pro-invasive growth factors with relevance to disease. To this end, we generated a specific fibroblast invasion signature, followed by functional validation of *in silico*-predicted upstream regulators of fibroblast invasion; namely transforming growth factor (TGF)β1, epidermal growth factor (EGF), fibroblast growth factor 2 (FGF2), and platelet-derived growth factor (PDGF)-BB. Prospectively, this validated fibroblast invasion signature will enable the identification of yet unknown invasion-relevant molecular networks and underlying mechanisms thereof. Thus, this invasion signature advances the understanding of an activated invasive fibroblast phenotype in particular for fibrotic and metastatic pathologies.

## Results Section

### Deciphering a transcriptomic signature for invading fibroblasts

**Figure 1:**
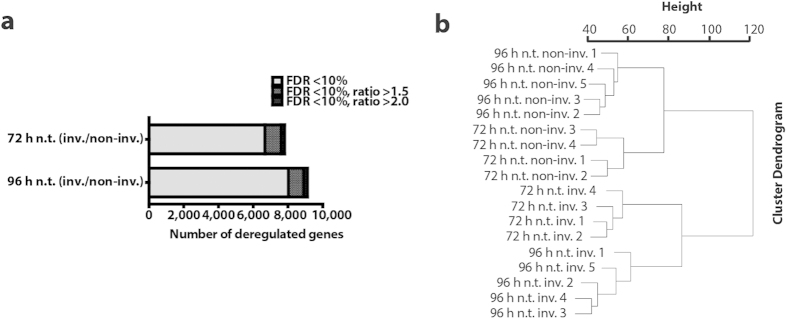
Analysis of differentially regulated targets of invading vs non-invading fibroblasts and hierarchical clustering. After 72 hours of invasion, 1,086 targets with expression ratios greater than 1.5-fold and 163 targets regulated greater than 2-fold were identified in the invading fraction. After 96 hours, an altered regulation of 1,049 probes with expression ratios greater than 1.5-fold and 182 greater than 2-fold were identified. The FDR for all of these targets was lower than 10%. The proportion of genes with expression ratios of 1 <> 1.5 between invading (inv.) and non-invading (non-inv.) phenotypes are depicted in light grey. The amount of targets with an expression ratio of >1.5 and >2 is represented in dark grey and black, respectively **(a)**. Hierarchical clustering distinguished two main clusters: invading (inv.) and non-invading (non-inv.) and two sub-clusters: 72 and 96 hours. The cluster dendrogram was conducted with R using the script hclust, on RMA data filtered for expression values >100 in at least one sample **(b)**.

**Figure 2:**
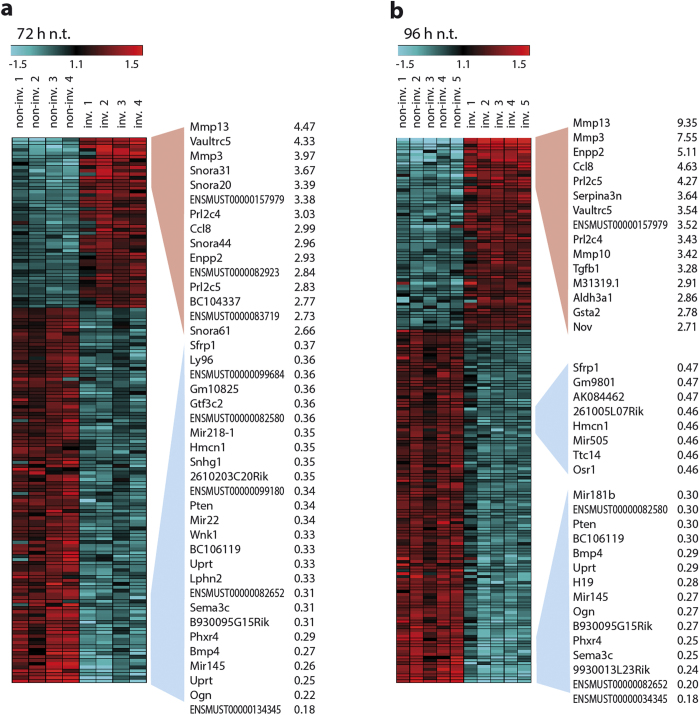
Transcriptome analysis (Affymetrix mouse gene ST 1.0 array) of fibroblast invasion. Separation and cell-lysis of MLg fibroblasts was performed 72 **(a)** and 96 hours **(b)** after plating the cells on top of the 3D collagen matrix. The displayed heatmaps show the top significantly up-, and down-regulated genes (>2-fold) that were found in the microarray analyses. Low and high expressed targets in the heatmap are depicted in blue and red, respectively. Each column represents one independent experiment. (n.t. = non-treated; non-inv. = non-invading fibroblasts; inv. = invading fibroblasts).

In order to systematically identify the molecular signature of invading fibroblasts we applied a whole transcriptome analysis (Affymetrix Mouse Gene 1.0 ST array), using our recently established 3D invasion assay^19^. A subpopulation of murine MLg 2908 (MLg) lung fibroblasts spontaneously invaded a 3D collagen matrix when plated on top of it^19^. Separation of invading from non-invading MLg fibroblasts was carried out 72 or 96 hours after plating. RNA isolation and subsequent whole transcriptome analysis provided gene expression profiles of invading and non-invading fibroblasts. Transcripts with a false discovery rate <10% were considered as being statistically significant regulated and were used for subsequent analyses. After 72 hours of invasion, 1,086 targets with expression ratios greater than 1.5-fold and 163 targets regulated greater than 2-fold were identified in the invading fraction as compared to the non-invading fraction. In fibroblasts that invaded the matrix for 96 hours, an altered regulation of 1,049 probes with expression ratios greater than 1.5-fold and 182 greater than 2-fold were identified (Fig. 1A). Hierarchical clustering of the different fractions is depicted in Fig. 1B. Heatmaps of up- and down-regulated target genes (>2-fold) in the invading fractions at 72 hours and 96 hours validate the reproducibility of our replicate analyses (Fig. 2A and Fig. 2B). The whole array data have been submitted to GEO (GSE55322).

### Predictive *in silico* cluster analysis and validation of the gene expression profile of fibroblast invasion

**Figure 3:**
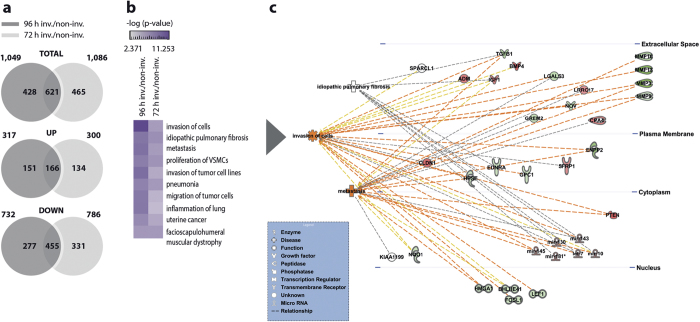
A time-dependent gene expression overlap of the conducted microarrays at 72 and 96 hours and predictive *in silico* analysis. Venn diagrams depict the expression overlap in deregulated genes (>1.5x) comparing the expression ratios of invading (inv.) and non-invading (non-inv.) fibroblasts at 96 hours and 72 hours after invasion. Note, 621 targets were found to overlap which comprise 166 up- and 455 down-regulated genes **(a)**. An IPA generated heatmap that shows the ten most significantly over-represented ‘disease processes’ and ‘biological functions’ including ‘invasion of cells’, ‘idiopathic pulmonary fibrosis’, and ‘metastasis’ **(b)**. Causal network analysis of underlying pathways of ‘invasion of cells’, ‘idiopathic pulmonary fibrosis’, and ‘metastasis’. Targets that were significantly up-, or down-regulated in the invading fibroblast phenotype are represented in green and red, respectively. The dashed orange lines illustrate activating relationships, yellow lines point out findings that are inconsistent with the state of downstream molecules, and grey lines indicate that the mode of effect is not defined **(c)**.

**Figure 4:**
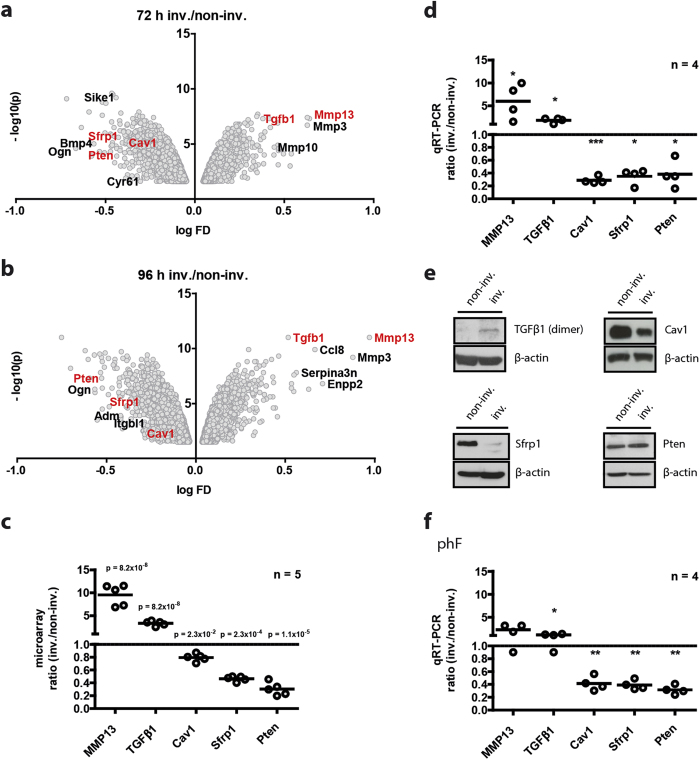
Expression levels of selected genes from the microarray analyses and validation by qRT-PCR and immunoblotting. Volcano plots depict the significantly differentially regulated probesets in the invading fraction at 72 **(a)** and 96 hours **(b)** time-points. Targets, used for microarray verification by qRT-PCR are highlighted in red. Here, the expression ratios derived from the microarray data are shown for a selected group of genes reportedly known to be functionally involved in the invasion of cells. The graph depicts the up-regulated targets in the invading fraction that is MMP13 (9.5x) and TGFβ1 (3.3x), as well as the down-regulated targets Cav1 (0.8x), Pten (0.3x) and Sfrp1 (0.5x) (n = 5). Data are shown with Benjamini-Hochberg (BH)-adjusted p-values. Genewise testing for differential expression employed the limma t-test and Benjamini-Hochberg multiple testing correction (FDR < 10%) **(c)**. qRT-PCR analyses confirming the differential expression of a selected group of genes (MMP13, TGFβ1, Cav1, and Pten) with the differential expression data derived from the microarrays. The data represent the fold induction values of the ratio between invading and non-invading fibroblasts. Data are shown as mean values from four independent experiments (n = 4). Statistical analysis: paired t-test. *p < 0.05 and ***p < 0.001 **(d)**. Representative immunoblots of TGFβ1, Cav1, Sfrp1 and Pten in invading (inv.) and non-invading (non-inv.) mouse lung fibroblasts on protein level. Immunoblots were cropped to improve clarity. Uncut blots are depicted in supplementary Fig. S2 **(e)** in invading (inv.) and non-invading (non-inv.) mouse lung fibroblasts. qRT-PCR analyses in inv. primary human fibroblasts (phF) reveals a similar deregulation of MMP13, TGFβ1, Cav1, Sfrp1 and Pten as found in the MLg fibroblasts **(f)**. Data shown represent mean values from four independent experiments (n = 4). Statistical analysis: paired t-test. *p < 0.05 and **p < 0.01.

The gene expression profiles of the invading fibroblasts at 72 hours and 96 hours (>1.5-fold) greatly overlapped: among the differentially regulated genes in the invading subtype at 72 and 96 hours, 621 genes overlapped in total: 166 in the up- and 455 in the down-regulated group (Fig. 3A). Of note, there were more than twice as many overlapping down-regulated genes than overlapping up-regulated genes. This comparative approach allowed us to enrich for those targets that are commonly regulated after 72 and 96 hours of invasion and to define the invasion signature of fibroblasts. Enrichment analyses using IPA’s ‘disease and function’ ontology revealed that ‘invasion of cells’, ‘idiopathic pulmonary fibrosis (IPF)’, and ‘metastasis’ ranked as the top three most significantly over-represented ‘disease processes’ and ‘biological functions’ within the invasion signature (Fig. 3B). In agreement with the well-known role of TGFβ1 in invasion and fibrosis, TGFβ1 associated with all three key networks of invasion (Fig. 3C). These data clearly corroborate our experimental approach. In order to further validate the profiling approach used, several known invasion-promoting genes were chosen for confirmative expression analysis by qRT-PCR. These targets included matrix metalloprotease 13 (MMP13)^20^,^21^, TGFβ1^10^,^22^, Caveolin 1 (Cav1)^23^, secreted frizzled-related protein (Sfrp) 1 24, and Phosphatase and Tensin Homolog (Pten)^25^,^26^ (Fig. 4A,B). Consistent with our microarray data (Fig. 4C), qRT-PCR analysis demonstrated a significant up-regulation of MMP13 and TGFβ1 in the invading fibroblasts by 6- and 1.8-fold, respectively (Fig. 4D). By contrast, Cav1, Sfrp1, and Pten were significantly down-regulated by 3.5-, 2.9-, and 2.6-fold, respectively (Fig. 3D). Reduced Sfrp1 and Cav1 protein expression was further corroborated by immunoblot analysis (Fig. 4E). Furthermore, TGFβ1 and MMP13^19^ were up-regulated on protein level as well (Fig. 4E). Interestingly, the protein expression of Pten, which was strongly diminished on transcript level, was found to be largely unchanged. Additionally, in primary human lung fibroblasts we identified a corresponding regulation of the same target genes in the invading subpopulation (Fig. 4F). The qRT-PCR analyses of selected target genes thus validated the robustness of our invasion assay and the identified gene signature for invading fibroblasts.

### Identification and validation of upstream regulators for fibroblast invasion

**Figure 5:**
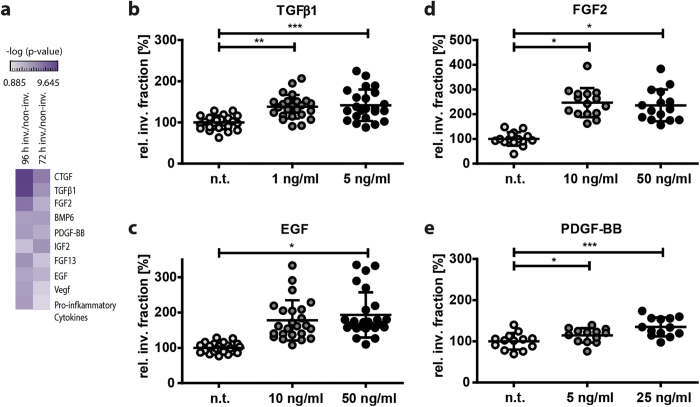
Causal network analysis, the identification and validation of pro-invasive growth-factors in the invasion signature of fibroblasts. A heatmap that indicates a ranking of physiological upstream regulators (filtered for ‘growth factors’) according to their p-values for gene expression data at 72 and 96 hours upon invasion (**a**). The predicted activated upstream regulators were functionally confirmed *in vitro* by a 3D invasion assay. Therefore, the invasive capacity of MLg fibroblasts was assessed by software-based quantification of the number of fibroblast nuclei (stained with DAPI) within and on top of the collagen matrix of 3D reconstructed confocal z-stacks. Fibroblasts were treated and left to invade the 3D collagen matrices for 48 hours. TGFβ1 (1 and 5 ng/ml), FGF2 (10 and 50 ng/ml), PDGF-BB (5 and 25 ng/ml) and EGF (10 and 50 ng/ml) were tested in the invasion assay (**b**-**e**). The data shown represent mean values (s.d.) from three to five independent experiment (n = 3–5) including five technical replicates each. (n.t. = non-treated). Statistical analysis: One way ANOVA with Dunnett’s multiple comparison test. *p < 0.05, **p < 0.01, and ***p < 0.001.

**Figure 6:**
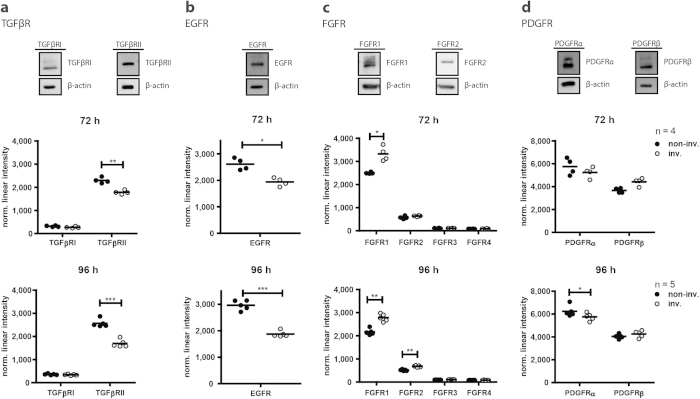
Expression levels of growth factor receptors. The baseline expression levels of transforming growth factor (TGF)β receptors I (TGFβRI) and II (TGFβRII), epidermal growth factor (EGF) receptor (EGFR), fibroblast growth factor (FGF) receptor 1 (FGFRI) and 2 (FGFR2), and platelet derived growth factor (PDGF) receptor alpha (PDGFRα) and beta (PDGFRβ) were determined by immunoblot analysis (**a–d** upper panel). Expression levels upon invasion at 72 hours (72 h) (**a–d** middle panel) and 96 hours (96 h) (**a–d** lower panel) were extracted from the microarray data. Statistical analysis: paired t-test. *p < 0.05, **p < 0.01, and ***p < 0.001.

In order to further validate the generated transcriptomic fibroblast invasion signature, gene expression lists were evaluated with the causal analysis tool ‘upstream regulator analysis’ (URA), implemented in IPA. This approach allowed the identification of putative upstream regulators for subsequent functional testing in regards to fibroblast invasion. In order to measure the enrichment of potentially activated upstream regulators we used a ranking based on the p-value^27^ obtained from the overlap between our experimentally derived gene datasets and those from the IPA’s database. As physiological upstream regulators were of particular interest, we excluded ‘drugs’, and ‘chemicals’ from the URA. The ‘p-value of overlap’ in supplementary Table 1 depicts a ranking of physiological upstream regulators according to their p-values for the comparison of gene expression data at 72 and 96 hours upon invasion. The cut-off value for the ranking (negative logarithmic p-value) was set to 4. By using a more stringent filter for ‘growth factors’, we were able to extract four growth factors, which all were found to be associated with fibrotic and/or cancerous diseases in the literature (Fig. 5A). Notably, TGFβ1, which is a prominent and potent activator of fibroblasts, ranked high among the predicted upstream regulators (p-value ^96 hours ^= 3.2 × 10^−10^, p-value ^72 hours ^= 1.4 × 10^−6^). In line with its key role in fibrogenesis, in the microarrays we found the expression of TGFβ1 to be significantly increased in the invading fibroblast fraction and moreover, also predicted to be associated with the generated functional gene clusters ‘IPF’, ‘invasion of cells’, and ‘metastasis’ in the *in silico* gene cluster analysis (Fig. 3C). Further putative upstream regulators from the URA, which were chosen for functional analysis in the following due to their association to fibrotic and cancerous diseases, comprised epidermal growth factor (EGF), basic fibroblast growth factor (FGF) 2, and platelet derived growth factor (PDGF)-BB. EGF, was predictively activated in the invading fibroblast fraction with a p-value ^96 hours ^= 10.0 × 10^−6^ and a p-value ^72 hours ^= 4.8 × 10^−5^ (Fig. 5A), while its receptor EGFR was also predicted to be activated (p-value ^96 hours ^= 6.3 × 10^−9^ and a p-value ^72 hours ^= 4.0 × 10^−5^) (supplementary Table 1). In our transcriptomic invasion signature, FGF2 was another highly ranked candidate in the predicted list of upstream regulators (p-value ^96 hours ^= 7.0 × 10^−8^ and p-value ^72 hours ^= 2.8 × 10^−5^), which was associated with fibrotic or cancerous diseases. Furthermore, PDGF-BB was predicted to be activated based on the invasion signature with p-value ^96 hours ^= 3.8 × 10^−6^ and a p-value ^72 hours ^= 2.3 × 10^−5^. Grounded on these computational predictions of activated upstream regulators, we next performed functional confirmative studies *in vitro*. Therefore, the invasive capacity of MLg fibroblasts was assessed by using the automated software-based invasion assay recently established in our lab^19^. The fibroblasts were treated with the particular growth factor and the invasion capacity was measured after 72 hours total invasion time as previously described^19^. TGFβ1 (1 and 5 ng/ml) significantly enhanced the fibroblast invasion capacity to 138.2 ± 28.7% (p-value ≤ 0.01) and 141.7 ± 38.1% (p-value ≤ 0.01), compared to untreated controls (Fig. 5B). EGF (10 and 50 ng/ml) significantly increased the cellular invasion to 178 ± 57.0% and 193.3 ± 64.2% (p-value ≤ 0.05), respectively (Fig. 5C). FGF2 exhibited the strongest significant effect on the invasion capacity with a mean relative induction of 246.8 ± 58.9% (10 ng/ml) (p-value ≤ 0.05) and 235.0 ± 64.2% (50 ng/ml) (p-value ≤ 0.05) (Fig. 5D). PDGF-BB (5 and 25 ng/ml) initiated a significant effect of 114.4 ± 17.6% (p-value ≤ 0.05) and 134.9 ± 23.0% (p-value ≤ 0.001) relative invasion (Fig. 5D). Supplementary Fig. S1 depicts representative images of either untreated or TGFβ1-, and FGF2-treated fibroblasts invading the collagen gel. Next, we used immunoblot analyses to demonstrate expression of the receptors for the above identified growth factors in lung fibroblasts. Protein expression of TGFβRI and II, EGFR, FGFR1 and 2, as well as PDGFRα and β was assessed in fibroblasts cultured on the 3D collagen matrix at the time of cell treatment. In addition, the expression levels of these receptors upon invasion were extracted from the microarray data for 72 and 96 hours of invasion (Fig. 6). While mRNA expression levels of TGFβRII and EGFR were detected but significantly reduced in invading cells at both time-points, FGFR1 and FGFR2 mRNA levels were increased. PDGFRα and β were expressed, but unchanged during invasion. Taken together, we have generated and identified, using a novel high content 3D invasion assay, a specific transcriptome signature of fibroblast invasion that shows a strong relation to invasive diseases, such as fibrosis and cancer.

## Discussion

The dynamic processes of tissue remodelling and repair that take place as a physiological response to tissue injury depend on an active and versatile fibroblast phenotype that ultimately restores normal tissue architecture and homeostasis^28^. Conversely, aberrantly activated fibroblasts are known to be involved in the initiation and progression of most malignant diseases such as cancer and fibrosis^6^,^16^,^29^. One substantial feature of activated fibroblasts is their increased invasive capacity^2^,^14^. The herein identified invasion signature included several genes that were previously reported to play essential roles for cellular invasion. In the expression profile of the invading fibroblast, the interstitial collagenase MMP13 and TGFβ1 were found to be significantly up-regulated while Cav1, Pten, and Sfrp1 were down-regulated on mRNA level. Finding MMP13 as one of the most highly up-regulated transcript in the invasion signature indicates that ECM degradation likely assists in the process of fibroblast invasion in the 3D invasion model. In a pathophysiological context, MMP13 expression in invading fibroblasts was found to not only drive their own invasion, but also influences the invasive capacity of carcinoma cells as indicated by MMP13 expression in subsets of activated carcinoma-associated fibroblasts (CAFs)^20^,^30^. Up-regulation of MMP13 together with MMP1 and MMP7^31^ in lung homogenates occurs in IPF, with the main cellular source of MMP13 being the lung epithelium and interstitial spaces^32^. Additionally, Cav1 and Pten, which were widely studied in the context of fibrosis and cancer, as well as cellular invasion, were included in our list of differentially expressed targets upon invasion^25^,^26^,^33^,^34^,^35^. Activated fibroblasts in IPF were reported to exhibit decreased Pten expression, correlating with increased αSMA levels^36^. Furthermore, α5β1 integrin-mediated invasion of lung fibroblasts was found to be circumvented through the reactivation of Pten activity by α4β1 integrin^25^. Pten also acts as stabilizer of junctional complexes in epithelial kidney cells, thereby preventing transformation to an invasive phenotype^26^. Furthermore, Pten was reported to suppress 2D cell migration and invasion in Boyden chamber *in vitro* assays in fibroblasts^37^,^38^. Interestingly, using our 3D invasion assay, protein expression of Pten did not correlate with the down-regulation of Pten on transcript level upon invasion, but remained unchanged when comparing invading with non-invading fibroblasts. This could advocate a more complex regulation of protein expression of Pten on various levels, including translation, protein stability and its turnover, or epigenetic mechanisms, all of which might be altered upon cellular invasion. Actually, the expression of Pten was reported to be regulated by miR-18a in a stiffness-dependent manner^39^, which could influence and alter its expression levels when using soft 3D matrices, such as collagen I. In IPF, down-regulation of Pten in fibroblastic foci was found to be accompanied by decreased Caveolin 1 (Cav1) levels. Diminished Cav1 thereby reduce membrane-associated Pten, which in turn favours the activation of PI3K/Akt signal pathway^40^. Furthermore, Sfrp1 was found to be down-regulated upon fibroblast invasion. Sfrps are a family of glycoproteins that can trigger Wnt signalling cascades by binding to Wnt ligands and Frizzled receptors^41^. Transcriptional silencing of Sfrp1 was previously reported in non-small cell lung cancer^42^ and in fibroblasts derived from keloid lesions^43^, Systemic Sclerosis (SSc)^44^, and IPF fibrotic lungs^45^. In breast cancer, ectopic expression of Sfrp1 in highly invasive human derived MDA-MB-231 adenocarcinoma cells decreased the migratory potential of the cells and impaired tumour outgrowth, and blocked lung metastases^24^. Reduction of Sfrp1 expression by TGFβ1 treatment with a concomitant increase in cell invasion and migration was reported in A549 human lung adenocarcinoma cell line^46^. We also identified TGFβ1, one of the best characterized profibrotic mediators, to be up-regulated in the invasion signature^47^. Here, TGFβ1 was found to be predictively associated with the functional clusters ‘invasion of cell’, ‘idiopathic pulmonary fibrosis (IPF)’, and ‘metastasis’. As the mere expression level of upstream regulators such as TGFβ1 was found to be significantly higher in invading fibroblasts, we aimed to validate the functional relevance of the generated invasion signature by using a systematic approach (Upstream Regulator Analysis (URA), Ingenuity). URA has frequently been used in the literature^48^,^49^,^50^. URA enables to elucidate upstream biological causes to gene-expression datasets and thus allows interpretation of gene expression data beyond gene cluster enrichment^27^. Using such causal networks in order to identify biological cause-effect relationships based on gene expression data is more powerful and relevant than looking at changes in gene expression levels alone^27^. The URA of differentially expressed genes comparing invading to non-invading fibroblasts provided us with an activation list of upstream regulators of various molecule types such as cytokines, chemical drugs, kinases, transcriptional regulators, growth factors, and others. The activation of the upstream regulators is entirely based on prediction grounded on the Ingenuity Knowledge Base, which is a large collection assembled from already published findings or third-party databases. Therefore, from the list of predictively activated upstream regulators, we functionally tested several cancer- and/or fibrosis-associated growth factors in our 3D invasion assay. Consistent with the URA prediction of our experimentally derived invasion signature, TGFβ1 induced fibroblast invasion. Taking into consideration that activated, invading fibroblasts in the 3D invasion model express higher levels of TGFβ1, this invading fibroblast phenotype may initiate the activation of adjuvant fibroblasts in a pathophysiological context. Besides TGFβ1, the identification and functional verification of additional cancer or fibrosis-associated factors, EGF, FGF2 and PDGF-BB, further confirmed the functional relevance of the invasion signature. In fibrotic and cancerous diseases, epidermal growth factor (EGF) represents one potent inducer of cellular invasion, which was also shown for breast and prostate cancer cells, oral carcinoma cells^51^, and human dermal fibroblasts^52^. Moreover, FGF2 was reported to be highly expressed in osteosarcoma-associated stromal cells^53^, and the expression of FGF2 was shown to be enhanced in renal fibrosis^54^ as well as in bronchoalveolar lavage fluid of IPF patients^55^. Furthermore, the inhibition of fibroblast growth factor receptor (FGFR) 1, the predominant receptor for FGF2, ameliorated hepatic fibrosis in a rodent model^56^. Another highly ranked candidate predicted to be an activated upstream regulator in our transcriptomic invasion signature was PDGF-BB. Most recently, PDGF-BB/PDGFR-β signalling was reported to contribute to fibrocyte migration in pulmonary fibrosis^57^. In addition, an involvement of this signalling cascade was suggested for cervical cancer^58^. Furthermore, it was suggested that cancer cells, secreting high amounts of PDGF activate adjuvant fibroblasts through a paracrine mechanism^59^. As of now the exact mechanisms that regulate fibroblast activation and their role in the initiation and progression of diseases such as fibrosis and cancer are not fully understood. Anyway, fibroblasts might be a potent target for therapeutic intervention. As such, the transcriptomic invasion signature of fibroblasts presented in our study describes the invading phenotype to unprecedented detail and provides a powerful repository for future studies on fibroblast invasiveness and the feasibility of defining therapeutic strategies in targeting activated fibroblasts. In particular, secreted proteins such as MMP13 or Sfrp1, may represent promising therapeutic targets in pathologies exhibiting fibroblast invasion. By systematically analysing the interactome of the components identified herein, we will be able to further refine the mechanistic processes involved in fibroblast activation and invasion.

## Material and Methods

### Antibodies

For immunoblotting the following primary (1) and secondary antibodies (2) were used: (1) caveolin 1 (anti-cav1 (D46G3) XP, mono-clonal, rabbit, Cell Signalling (Danvers, MA, USA), 1:1,000), EGFR (anti-EGFR (D38B1), mono-clonal, rabbit, Cell Signalling, 1:1,000), FGFRI (anti-FGFRI (Ab-154), poly-clonal, rabbit, Sigma, 1:200), FGFRII (anti-FGFRII (CD332), poly-clonal, rabbit, Thermo Scientific (Rockford, IL, USA), 1:1,000), PDGFRα (anti- PDGFRα (C-20), poly-clonal, rabbit, Santa Cruz (Dallas, TX, USA), 1:500), PDGFRβ (anti- PDGFRβ (958), poly-clonal, rabbit, Santa Cruz, 1:1,000), Pten (138G6), mono-clonal, rabbit, Cell Signalling, 1:1,000), Sfrp1 (anti-Sfrp1(EPR7003), mono-clonal, rabbit, Abcam (Cambridge, UK), 1:1,000), TGFβ1 (anti-TGFβ1 (ab9758), Abcam, poly-clonal, rabbit, 1:200), TGFβRI (anti- TGFβRI (H-100), poly-clonal, rabbit, Santa Cruz, 1:1,000), TGFβRII (anti- TGFβRII (D-2), mono-clonal, mouse, Santa Cruz, 1:1,000) and monoclonal mouse anti-β-Actin-Peroxidase (AC-15, Sigma, 1:10,000); (2) goat anti-rabbit and goat anti-mouse IgG conjugated to horseradish peroxidase (Cell Signalling, 1:10,000) as secondary antibody.

### Cell Culture

Mouse lung fibroblasts, MLg (MLg 2908) were purchased from ATCC (Manassas, VA, USA) (CCL-206) and cultivated in DMEM/HAM’s F12 (catalog# E15-813, PAA (Pasching, Austria)) medium containing 10% FBS (PAA). For isolation of primary human lung fibroblasts, specimens from lung lobes or segmental lung resections were dissected into pieces of 1–2 mm^2^ in size and digested by 5 mg of Collagenase I (Biochrom (Berlin, Germany)) at 37 °C for 2 hours. Subsequently, samples were filtered through nylon filters with a pore size of 70 μm (BD Falcon, (Bedford, MA, USA)). Filtrates, containing the cells, were centrifuged at 400 g, 4 °C for 5 minutes. Pellets were resuspended in DMEM/F-12 medium (Gibco, (Darmstadt, Germany)) supplemented with 20% fetal bovine serum (PAA) and plated on 10 cm cell-culture dishes and subsequently cultured to a confluence of 80–90% in DMEM/HAM’s F12 medium containing 20% FBS. All cells were cultivated under standard conditions (5% CO_2_ and 37 °C). MLg fibroblasts and primary cells were not used at passage numbers higher than 15 and 10, respectively.

### 3D Collagen Invasion Assay

3D cell culture assays were performed as previously described^19^. 2 × 10^4^ cells per well were seeded on top of the matrix and left for invasion under standard conditions (37 °C, 5% CO_2_) in DMEM/HAM’s F12 medium containing 5% FBS. Treatment of cells with recombinant epidermal growth factor (EGF) (Sigma), transforming growth factor beta-1 (TGFβ1) (R&D, Minneapolis, MN, USA)), fibroblast growth factor (FGF) 2 (R&D) or platelet derived growth factor (PDGF) BB (life technologies; Carlsbad, USA) was accomplished by treating cells with 10–50 ng/ml EGF and FGF2, 1–5 ng/ml TGFβ1, or 5–25 ng/ml PDGF-BB 24 hours after plating, and culturing them up to 72 hours in total.

### Separation Assay, Protein and mRNA Isolation from collagen-embedded Cells

The separation of invading from non-invading cells and the isolation of protein and mRNA was performed as previously described^19^. For immunoblot analysis of growth factor receptors, 5 × 10^5^ cells were plated on top of 1.5 ml polymerized collagen G (Biochrom) per well of a 6-well plate in DMEM/HAM’s F12 medium containing 5% FBS. Protein isolation was performed 24 hours after plating, as described before^19^.

### mRNA Isolation and qRT-PCR

mRNA from fibroblasts, cultured in the 3D cell culture model, was isolated as previously described^19^. Mouse primer sequences: GTGCGAGCCGGTCATGCAGT (Sfrp1_fw), CACACGGTTGTACCTTGGGGC (Sfrp1_rev), TGTGTCCGTCGTGGATCTGA (GAPDH_fw), CCTGCTTCACCACCTTCTTGA (GAPDH_rev), CGACGACGTGGTCAAGATTGACTTT (Cav1_fw), TGCACGGTACAACCGCCCAG (Cav1_rev), ATCCCTTGATGCCATTACCA (MMP13_fw), AAGAGCTCAGCCTCAACCTG (MMP13_rev), GTGGACCGCAACAACGCC (TGFβ1_fw), TGGGGGTCAGCAGCCGGT (TGFβ1_rev), TCAGTGGCGGAACTTGCAATCCT (Pten_fw), CGCCGCGTGGGTCCTGAAT (Pten_rev). Human primer sequences: GGACCGGCCCATCTACCCGT (Sfrp1_fw), ACACCGTTGTGCCTTGGGGC (Sfrp1_rev), TGACCTCAACTACATGGTTTACATG (GAPDH_fw), TTGATTTTGGAGGGATCTCG (GAPDH_rev), GGACATCTCTACACCGTTCCC (Cav1_fw), CTTGACCACGTCATCGTTGAG (Cav1_rev), AGGCTCCGAGAAATGCAGTC (MMP13_fw), ATCAGGAACCCCGCATCTTG (MMP13_rev), TTCGCCTTAGCGCCCACTGC (TGFβ1_fw), GGCCGGTAGTGAACCCGTTG (TGFβ1_rev), TGGCGGAACTTGCAATCCTCAGT (Pten_fw), TCCCGTCGTGTGGGTCCTGA (Pten_rev). cDNA was synthesized with the GeneAMP PCR kit (Applied Biosystems (Foster City, CA, USA)) utilizing random hexamers using 1 μg of isolated RNA for one reaction. Denaturation was performed in an Eppendorf Mastercycler with the following settings: lid = 45 °C, 70 °C for 10 minutes and 4 °C for 5 minutes. Reverse transcription was performed in an Eppendorf Mastercycler with the following settings: lid = 105 °C, 20 °C for 10 minutes, 42 °C for 60 minutes and 99 °C for 5 minutes. qRT-PCR reactions were performed in triplicates with SYBR Green I Master in a LightCycler® 480II (Roche (Risch, Switzerland)) with standard conditions: 95 °C for 5 min followed by 45 cycles of 95 °C for 5 s (denaturation), 59 °C for 5 s (annealing) and 72 °C for 20 s (elongation). Target genes were normalized to GAPDH expression.

### Microarray

For microarray analysis, total RNA was isolated with the miRNeasy mini kit® according to the manufacturer’s protocol (Qiagen (Hilden, Germany)). RNA quality was accessed by the Agilent 2100 Bioanalyzer and only high quality RNA (RIN > 8) was used for microarray analysis. Total RNA (150 ng) was amplified using the Ambion WT Expression Kit and the WT Terminal Labeling Kit (Affymetrix (Santa Clara, USA)). Amplified cDNA (2.75 μg) was hybridized on Affymetrix Mouse Gene 1.0 ST arrays containing about 28,000 probe sets. Staining (Fluidics script FS450_0007) and scanning was done according to the Affymetrix expression protocol. For the statistical transcriptome analysis, expression console (Affymetrix) was used for quality control and to obtain annotated normalized RMA gene-level data (standard settings including sketch-quantile normalisation). Array data has been submitted to GEO (GSE55322).

### Protein Isolation, SDS-PAGE and Western Blotting

Protein from fibroblasts, cultured in the 3D cell culture model, was isolated as previously described^19^. Samples were mixed with 50 mM Tris-HCl, pH 6.8, 100 mM DTT, 2% SDS, 1% bromphenol blue, and 10% glycerol, and proteins were separated using standard SDS-10% PAGE. For immunoblotting, proteins were transferred to PVDF (Millipore (Billerica, MA, (USA)), 0.45 μm or 0.2 μm) membranes, which were blocked with 5% milk in TBST (0.1% Tween 20/TBS) and incubated with primary, followed by HRP-conjugated secondary antibodies over night at 4 °C and at room temperature for 1 hour, respectively.

### *In silico* analysis

Regulated gene sets from the microarray data were analyzed through the use of IPA (Ingenuity® Systems, www.ingenuity.com). The ‘disease and function’ ontology was used to determine significantly enriched terms (p > 0.01, Right-tailed Fisher’s exact test). Upstream regulator activation analysis (URA) implemented in IPA was used to predict activators that are directly or indirectly connected to the dataset of genes. Upstream regulators with a p-value of overlap ≤ 0.0001 and a positive activation z-score at both timepoints were considered activated.

### Statistics

Statistical analysis was performed using GraphPad Prism4 (GraphPad Software). Data are presented as mean ± s.d. Statistical analysis was performed using unpaired and paired t-tests (two-tailed) or One way ANOVA with Dunnett’s multiple comparison test. For microarray experiments statistical analyses were performed by utilizing the statistical programming environment R implemented in CARMAweb^60^. Genewise testing for differential expression was done employing limma t-test and Benjamini-Hochberg multiple testing correction (FDR < 10%). Right-tailed Fisher Exact Test was utilized for statistical analysis on the Ingenuity Pathways Analysis (IPA) platform (Ingenuity Systems (Redwood City, CA, USA)).

## Additional Information

**How to cite this article**: Oehrle, B. *et al.* Validated prediction of pro-invasive growth factors using a transcriptome-wide invasion signature derived from a complex 3D invasion assay. *Sci. Rep.* **5**, 12673; doi: 10.1038/srep12673 (2015).

**Accession codes**: GEO (GSE55322)

## Supplementary Material

Supplementary Information

## References

[b1] Lauffenburger D. A. & Horwitz A. F. Cell migration: a physically integrated molecular process. Cell 84, 359–369 (1996). 8608589 10.1016/s0092-8674(00)81280-5

[b2] Kalluri R. & Zeisberg M. Fibroblasts in cancer. Nat Rev Cancer 6, 392–401 (2006). 16572188 10.1038/nrc1877

[b3] Bhowmick N. A., Neilson E. G. & Moses H. L. Stromal fibroblasts in cancer initiation and progression. Nature 432, 332–337 (2004). 15549095 10.1038/nature03096 PMC3050735

[b4] Friedl P., Zanker K. S. & Brocker E. B. Cell migration strategies in 3-D extracellular matrix: differences in morphology, cell matrix interactions, and integrin function. Microsc Res Tech 43, 369–378 (1998). 9858334 10.1002/(SICI)1097-0029(19981201)43:5<369::AID-JEMT3>3.0.CO;2-6

[b5] Schmidt S. & Friedl P. Interstitial cell migration: integrin-dependent and alternative adhesion mechanisms. Cell Tissue Res 339, 83–92 (2010). 19921267 10.1007/s00441-009-0892-9 PMC2784868

[b6] Klingberg F., Hinz B. & White E. S. The myofibroblast matrix: implications for tissue repair and fibrosis. J Pathol 229, 298–309 (2013). 22996908 10.1002/path.4104 PMC4005341

[b7] Hardie W. D., Glasser S. W. & Hagood J. S. Emerging concepts in the pathogenesis of lung fibrosis. Am J Pathol 175, 3–16 (2009). 19497999 10.2353/ajpath.2009.081170 PMC2708789

[b8] Cool C. D. *et al.* Fibroblast foci are not discrete sites of lung injury or repair: the fibroblast reticulum. Am J Respir Crit Care Med 174, 654–658 (2006). 16799077 10.1164/rccm.200602-205OC PMC2648056

[b9] Nasreen N. *et al.* Pleural mesothelial cell transformation into myofibroblasts and haptotactic migration in response to TGF-beta1 *in vitro*. Am J Physiol Lung Cell Mol Physiol 297, L115–124 (2009). 19411308 10.1152/ajplung.90587.2008 PMC2711818

[b10] Zolak J. S. *et al.* Pleural mesothelial cell differentiation and invasion in fibrogenic lung injury. Am J Pathol 182, 1239–1247 (2013). 23399488 10.1016/j.ajpath.2012.12.030 PMC3620419

[b11] Laurent G. J., McAnulty R. J., Hill M. & Chambers R. Escape from the matrix: multiple mechanisms for fibroblast activation in pulmonary fibrosis. Proc Am Thorac Soc 5, 311–315 (2008). 18403325 10.1513/pats.200710-159DR PMC2643217

[b12] Willis B. C. & Borok Z. TGF-beta-induced EMT: mechanisms and implications for fibrotic lung disease. Am J Physiol Lung Cell Mol Physiol 293, L525–534 (2007). 17631612 10.1152/ajplung.00163.2007

[b13] Coker R. K. & Laurent G. J. Pulmonary fibrosis: cytokines in the balance. Eur Respir J 11, 1218–1221 (1998). 9657557 10.1183/09031936.98.11061218

[b14] Li Y. *et al.* Severe lung fibrosis requires an invasive fibroblast phenotype regulated by hyaluronan and CD44. J Exp Med 208, 1459–1471 (2011). 21708929 10.1084/jem.20102510 PMC3135364

[b15] Dienus K., Bayat A., Gilmore B. F. & Seifert O. Increased expression of fibroblast activation protein-alpha in keloid fibroblasts: implications for development of a novel treatment option. Arch Dermatol Res 302, 725–731 (2010). 20872224 10.1007/s00403-010-1084-x

[b16] Hanahan D. & Weinberg R. A. Hallmarks of cancer: the next generation. Cell 144, 646–674 (2011). 21376230 10.1016/j.cell.2011.02.013

[b17] Otranto M. *et al.* The role of the myofibroblast in tumor stroma remodeling. Cell Adh Migr 6, 203–219 (2012). 22568985 10.4161/cam.20377 PMC3427235

[b18] Gaggioli C. *et al.* Fibroblast-led collective invasion of carcinoma cells with differing roles for RhoGTPases in leading and following cells. Nat Cell Biol 9, 1392–1400 (2007). 18037882 10.1038/ncb1658

[b19] Burgstaller G., Oehrle B., Koch I., Lindner M. & Eickelberg O. Multiplex profiling of cellular invasion in 3D cell culture models. PLoS One 8, e63121 (2013). 23671660 10.1371/journal.pone.0063121 PMC3650046

[b20] Lederle W. *et al.* MMP13 as a stromal mediator in controlling persistent angiogenesis in skin carcinoma. Carcinogenesis 31, 1175–1184 (2010). 19892798 10.1093/carcin/bgp248 PMC2893794

[b21] Sabeh F., Li X. Y., Saunders T. L., Rowe R. G. & Weiss S. J. Secreted versus membrane-anchored collagenases: relative roles in fibroblast-dependent collagenolysis and invasion. J Biol Chem 284, 23001–23011 (2009). 19542530 10.1074/jbc.M109.002808 PMC2755707

[b22] Imai K. *et al.* Bronchioloalveolar invasion in non-small cell lung cancer is associated with expression of transforming growth factor-beta1. World J Surg Oncol 11, 113 (2013). 23705641 10.1186/1477-7819-11-113 PMC3664590

[b23] Lino Cardenas C. L. *et al.* miR-199a-5p Is upregulated during fibrogenic response to tissue injury and mediates TGFbeta-induced lung fibroblast activation by targeting caveolin-1. PLoS Genet 9, e1003291 (2013). 23459460 10.1371/journal.pgen.1003291 PMC3573122

[b24] Matsuda Y., Schlange T., Oakeley E. J., Boulay A. & Hynes N. E. WNT signaling enhances breast cancer cell motility and blockade of the WNT pathway by sFRP1 suppresses MDA-MB-231 xenograft growth. Breast Cancer Res 11, R32 (2009). 19473496 10.1186/bcr2317 PMC2716500

[b25] White E. S. *et al.* Integrin alpha4beta1 regulates migration across basement membranes by lung fibroblasts: a role for phosphatase and tensin homologue deleted on chromosome 10. Am J Respir Crit Care Med 168, 436–442 (2003). 12791582 10.1164/rccm.200301-041OC PMC1997294

[b26] Kotelevets L. *et al.* Implication of the MAGI-1b/PTEN signalosome in stabilization of adherens junctions and suppression of invasiveness. Faseb j 19, 115–117 (2005). 15629897 10.1096/fj.04-1942fje

[b27] Kramer A., Green J., Pollard J. Jr. & Tugendreich S. Causal analysis approaches in Ingenuity Pathway Analysis. Bioinformatics 30, 523–530 (2014). 24336805 10.1093/bioinformatics/btt703 PMC3928520

[b28] Hinz B. *et al.* Recent developments in myofibroblast biology: paradigms for connective tissue remodeling. Am J Pathol 180, 1340–1355 (2012). 22387320 10.1016/j.ajpath.2012.02.004 PMC3640252

[b29] Crestani B., Besnard V., Plantier L., Borensztajn K. & Mailleux A. Fibroblasts: the missing link between fibrotic lung diseases of different etiologies? Respir Res 14, 81 (2013). 23915374 10.1186/1465-9921-14-81 PMC3734215

[b30] Lecomte J. *et al.* Bone marrow-derived myofibroblasts are the providers of pro-invasive matrix metalloproteinase 13 in primary tumor. Neoplasia 14, 943–951 (2012). 23097628 10.1593/neo.121092 PMC3479839

[b31] Rosas I. O. *et al.* MMP1 and MMP7 as potential peripheral blood biomarkers in idiopathic pulmonary fibrosis. PLoS Med 5, e93 (2008). 18447576 10.1371/journal.pmed.0050093 PMC2346504

[b32] Nkyimbeng T. *et al.* Pivotal role of matrix metalloproteinase 13 in extracellular matrix turnover in idiopathic pulmonary fibrosis. PLoS One 8, e73279 (2013). 24023851 10.1371/journal.pone.0073279 PMC3759404

[b33] Wang X. M. *et al.* Caveolin-1: a critical regulator of lung fibrosis in idiopathic pulmonary fibrosis. J Exp Med 203, 2895–2906 (2006). 17178917 10.1084/jem.20061536 PMC1850940

[b34] Goetz J. G. *et al.* Biomechanical remodeling of the microenvironment by stromal caveolin-1 favors tumor invasion and metastasis. Cell 146, 148–163 (2011). 21729786 10.1016/j.cell.2011.05.040 PMC3244213

[b35] Tamura M. *et al.* Inhibition of cell migration, spreading, and focal adhesions by tumor suppressor PTEN. Science 280, 1614–1617 (1998). 9616126 10.1126/science.280.5369.1614

[b36] White E. S. *et al.* Negative regulation of myofibroblast differentiation by PTEN (Phosphatase and Tensin Homolog Deleted on chromosome 10). Am J Respir Crit Care Med 173, 112–121 (2006). 16179636 10.1164/rccm.200507-1058OC PMC1434700

[b37] Liliental J. *et al.* Genetic deletion of the Pten tumor suppressor gene promotes cell motility by activation of Rac1 and Cdc42 GTPases. Curr Biol 10, 401–404 (2000). 10753747 10.1016/s0960-9822(00)00417-6

[b38] Tamura M., Gu J., Takino T. & Yamada K. M. Tumor suppressor PTEN inhibition of cell invasion, migration, and growth: differential involvement of focal adhesion kinase and p130Cas. Cancer Res 59, 442–449 (1999). 9927060

[b39] Mouw J. K. *et al.* Tissue mechanics modulate microRNA-dependent PTEN expression to regulate malignant progression. 20, 360–367 (2014). 10.1038/nm.3497 PMC3981899 24633304

[b40] Xia H. *et al.* Pathologic caveolin-1 regulation of PTEN in idiopathic pulmonary fibrosis. Am J Pathol 176, 2626–2637 (2010). 20395445 10.2353/ajpath.2010.091117 PMC2877826

[b41] Esteve P. & Bovolenta P. The advantages and disadvantages of sfrp1 and sfrp2 expression in pathological events. Tohoku J Exp Med 221, 11–17 (2010). 20448436 10.1620/tjem.221.11

[b42] Fukui T. *et al.* Transcriptional silencing of secreted frizzled related protein 1 (SFRP 1) by promoter hypermethylation in non-small-cell lung cancer. Oncogene 24, 6323–6327 (2005). 16007200 10.1038/sj.onc.1208777

[b43] Russell S. B. *et al.* Epigenetically altered wound healing in keloid fibroblasts. J Invest Dermatol 130, 2489–2496 (2010). 20555348 10.1038/jid.2010.162 PMC2939920

[b44] Dees C. *et al.* The Wnt antagonists DKK1 and SFRP1 are downregulated by promoter hypermethylation in systemic sclerosis. Ann Rheum Dis 73, 1232–1239 (2014). 23698475 10.1136/annrheumdis-2012-203194

[b45] Hsu E. *et al.* Lung tissues in patients with systemic sclerosis have gene expression patterns unique to pulmonary fibrosis and pulmonary hypertension. Arthritis Rheum 63, 783–794 (2011). 21360508 10.1002/art.30159 PMC3139818

[b46] Ren J. *et al.* sFRP1 inhibits epithelial-mesenchymal transition in A549 human lung adenocarcinoma cell line. Cancer Biother Radiopharm 28, 565–571 (2013). 23802127 10.1089/cbr.2012.1453 PMC3741431

[b47] Fernandez I. E. & Eickelberg O. The impact of TGF-beta on lung fibrosis: from targeting to biomarkers. Proc Am Thorac Soc 9, 111–116 (2012). 22802283 10.1513/pats.201203-023AW

[b48] Ampe C., Libbrecht J. & Van Troys M. beta-Actin knock-out mouse embryonic fibroblasts show increased expression of LIM-, CH-, EFh-domain containing proteins with predicted common upstream regulators. Cytoskeleton (Hoboken) 70, 766–774 (2013). 24123846 10.1002/cm.21147

[b49] Meier-Abt F. *et al.* Parity induces differentiation and reduces Wnt/Notch signaling ratio and proliferation potential of basal stem/progenitor cells isolated from mouse mammary epithelium. Breast Cancer Res 15, R36 (2013). 23621987 10.1186/bcr3419 PMC3672662

[b50] Sachs Z. *et al.* NRASG12V oncogene facilitates self-renewal in a murine model of acute myelogenous leukemia. Blood 124, 3274–3283 (2014). 25316678 10.1182/blood-2013-08-521708 PMC4239336

[b51] Lu Z., Jiang G., Blume-Jensen P. & Hunter T. Epidermal growth factor-induced tumor cell invasion and metastasis initiated by dephosphorylation and downregulation of focal adhesion kinase. Mol Cell Biol 21, 4016–4031 (2001). 11359909 10.1128/MCB.21.12.4016-4031.2001 PMC87064

[b52] Gobin A. S. & West J. L. Effects of epidermal growth factor on fibroblast migration through biomimetic hydrogels. Biotechnol Prog 19, 1781–1785 (2003). 14656156 10.1021/bp0341390

[b53] Shimizu T. *et al.* Fibroblast growth factor-2 is an important factor that maintains cellular immaturity and contributes to aggressiveness of osteosarcoma. Mol Cancer Res 10, 454–468 (2012). 22228819 10.1158/1541-7786.MCR-11-0347

[b54] Strutz F. *et al.* TGF-beta 1 induces proliferation in human renal fibroblasts via induction of basic fibroblast growth factor (FGF-2). Kidney Int 59, 579–592 (2001). 11168939 10.1046/j.1523-1755.2001.059002579.x

[b55] Inoue Y., King T. E. Jr., Tinkle S. S., Dockstader K. & Newman L. S. Human mast cell basic fibroblast growth factor in pulmonary fibrotic disorders. Am J Pathol 149, 2037–2054 (1996). 8952537 PMC1865345

[b56] Lin N. *et al.* NP603, a novel and potent inhibitor of FGFR1 tyrosine kinase, inhibits hepatic stellate cell proliferation and ameliorates hepatic fibrosis in rats. Am J Physiol Cell Physiol 301, C469–477 (2011). 21543745 10.1152/ajpcell.00452.2010

[b57] Aono Y. *et al.* Role of platelet-derived growth factor/platelet-derived growth factor receptor axis in the trafficking of circulating fibrocytes in pulmonary fibrosis. Am J Respir Cell Mol Biol 51, 793–801 (2014). 24885373 10.1165/rcmb.2013-0455OC

[b58] Tudoran O. M. *et al.* PDGF beta targeting in cervical cancer cells suggest a fine-tuning of compensatory signalling pathways to sustain tumourigenic stimulation. J Cell Mol Med (2014). 10.1111/jcmm.12449 PMC4407607 25311137

[b59] Forsberg K., Valyi-Nagy I., Heldin C. H., Herlyn M. & Westermark B. Platelet-derived growth factor (PDGF) in oncogenesis: development of a vascular connective tissue stroma in xenotransplanted human melanoma producing PDGF-BB. Proc Natl Acad Sci U S A 90, 393–397 (1993). 8380638 10.1073/pnas.90.2.393 PMC45668

[b60] Rainer J., Sanchez-Cabo F., Stocker G., Sturn A. & Trajanoski Z. CARMAweb: comprehensive R- and bioconductor-based web service for microarray data analysis. Nucleic Acids Res 34, W498–503 (2006). 16845058 10.1093/nar/gkl038 PMC1538903

