# Supplementary material for: Validated prediction of pro-invasive growth factors using a transcriptome-wide invasion signature derived from a complex 3D invasion assay
Source: Sci Rep. 2015 Aug 5;5:12673. doi: 10.1038/srep12673 (PMC4525140; doi:10.1038/srep12673)

**ONLINE DATA SUPPLEMENT**

**Validated prediction of pro-invasive growth factors using a transcriptome-wide invasion signature derived from a complex 3D invasion assay**

**Bettina Oehrle^1^, Gerald Burgstaller^1^, Martin Irmler^2^, Stefan Dehmel^1^, Jessica Grün^1^, Tiffany Hwang^1^, Susanne Krauss-Etschmann^1^, Johannes Beckers^2,3^, Silke Meiners^1^, Oliver Eickelberg^1,*^**

**^1^**Comprehensive Pneumology Center, University Hospital of the Ludwig-Maximilians-University Munich and Helmholtz Zentrum München, Member of the German Center for Lung Research, 81377 Munich, Germany, ^2^Institute of Experimental Genetics, Helmholtz Zentrum München, German Research Center for Environmental Health (GmbH), 85764 Neuherberg, Germany, ^3^Technical University Munich, Chair of Experimental Genetics, 85350 Freising-Weihenstephan, Germany

*To whom correspondence should be addressed: Oliver Eickelberg, Comprehensive Pneumology Center, Ludwig-Maximilians-Universität and Helmholtz Zentrum München, Max-Lebsche-Platz 31, 81377 München, Germany, Tel.: 0049(89)31874666; Fax: 0049(89)31874661; Email: [oliver.eickelberg@helmholtz-muenchen.de](mailto:oliver.eickelberg@helmholtz-muenchen.de)

**SUPPLEMENTARY TABLE**

**Table S1: Identification of upstream regulators for the invasion signature in fibroblasts.** Using URA implemented in IPA, a ranking of the most significant biological upstream regulators for the transcriptome invasion signature was conducted. The clusters ‘chemicals’ and ‘drugs’ were excluded from the analysis. A cut-off for the negative p-value of 4 was set and data underneath this cut-off are presented in grey.

**
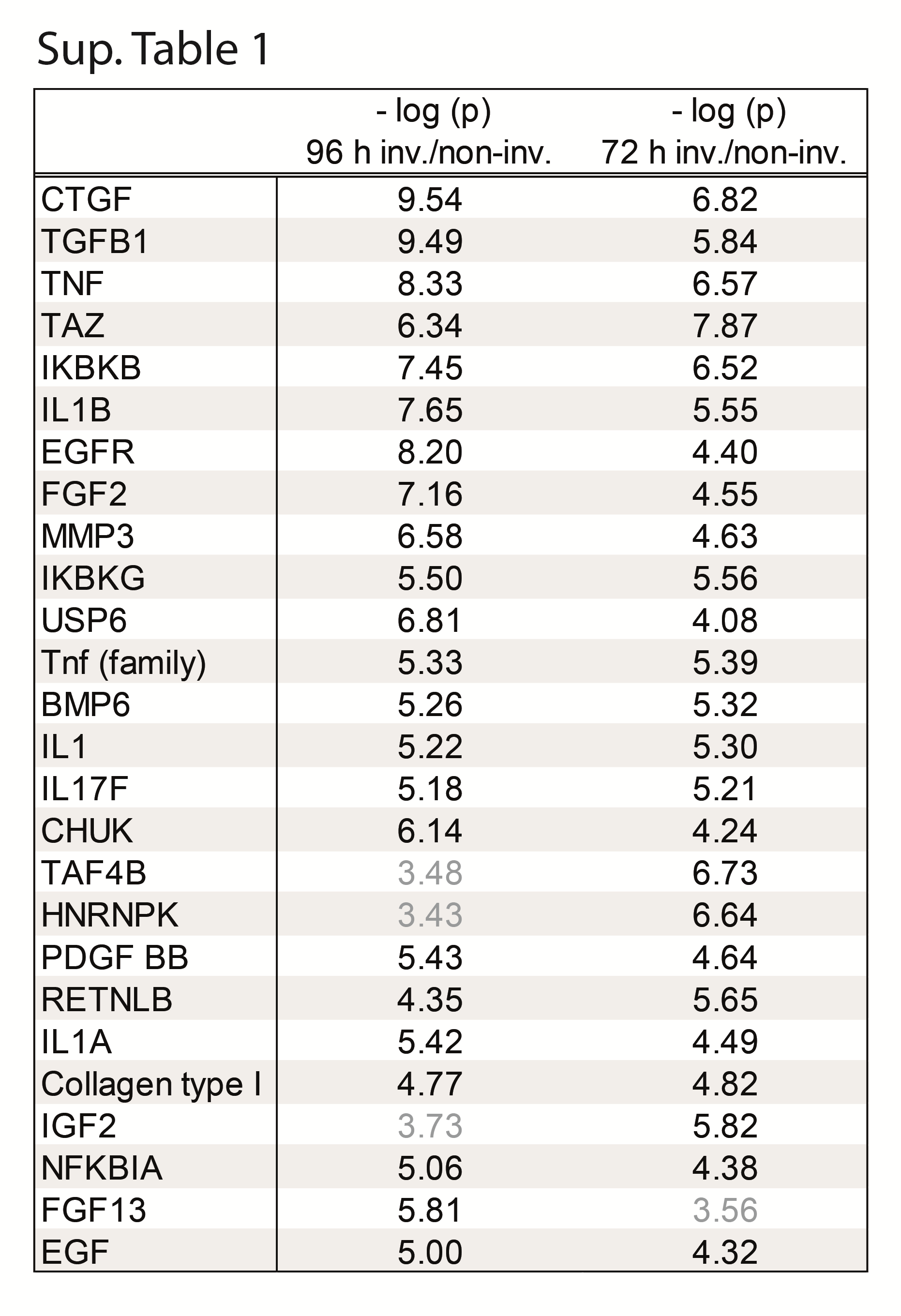
**

**SUPPLEMENTARY FIGURE LEGEND**

***Supplementary Figure S1.* TGFβ1 and FGF2 increase the invasive capacity of lung fibroblasts.** The invasive capacity of MLg fibroblasts was assessed by automated software-based quantification of the number of fibroblast nuclei (stained with DAPI) within and on top of the collagen matrix of 3D reconstructed confocal z-stacks. Fibroblasts were treated with TGFβ1 (1 – 5 ng/ml), or FGF2 (10 – 50 ng/ml) and left to invade the 3D collagen matrices for 48 hours. Invasion depth ranged between 0-700 µm, colour coding from red to yellow, bottom to top. Scale bar 300 µm.

***Supplementary Figure S2.* Validation of Cav1, Sfrp1, Pten and TGFβ1 regulation in invading fibroblast by immunoblotting.**

Full-length blots to Figure 4E of Caveolin1 (Cav1), secreted frizzled-related protein1 (Sfrp1), transforming growth factor (TGF)β1, Phosphatase and Tensin Homolog (Pten), and β-actin as a loading control are shown. Protein samples were probed on 12% - 15% gels using the same experimental conditions. Signals were captured on X-ray film by developing using the AGFA Curix 60 processor. For Sfrp1, recombinant human Sfrp1 (rh-Sfrp1) was used as control. For Pten and TGFβ1 knockdown (kd) experiments with the respective siRNA were performed. Unspecific bands are highlighted with an asterisk.

**SUPPLEMENTARY FIGURES**

**
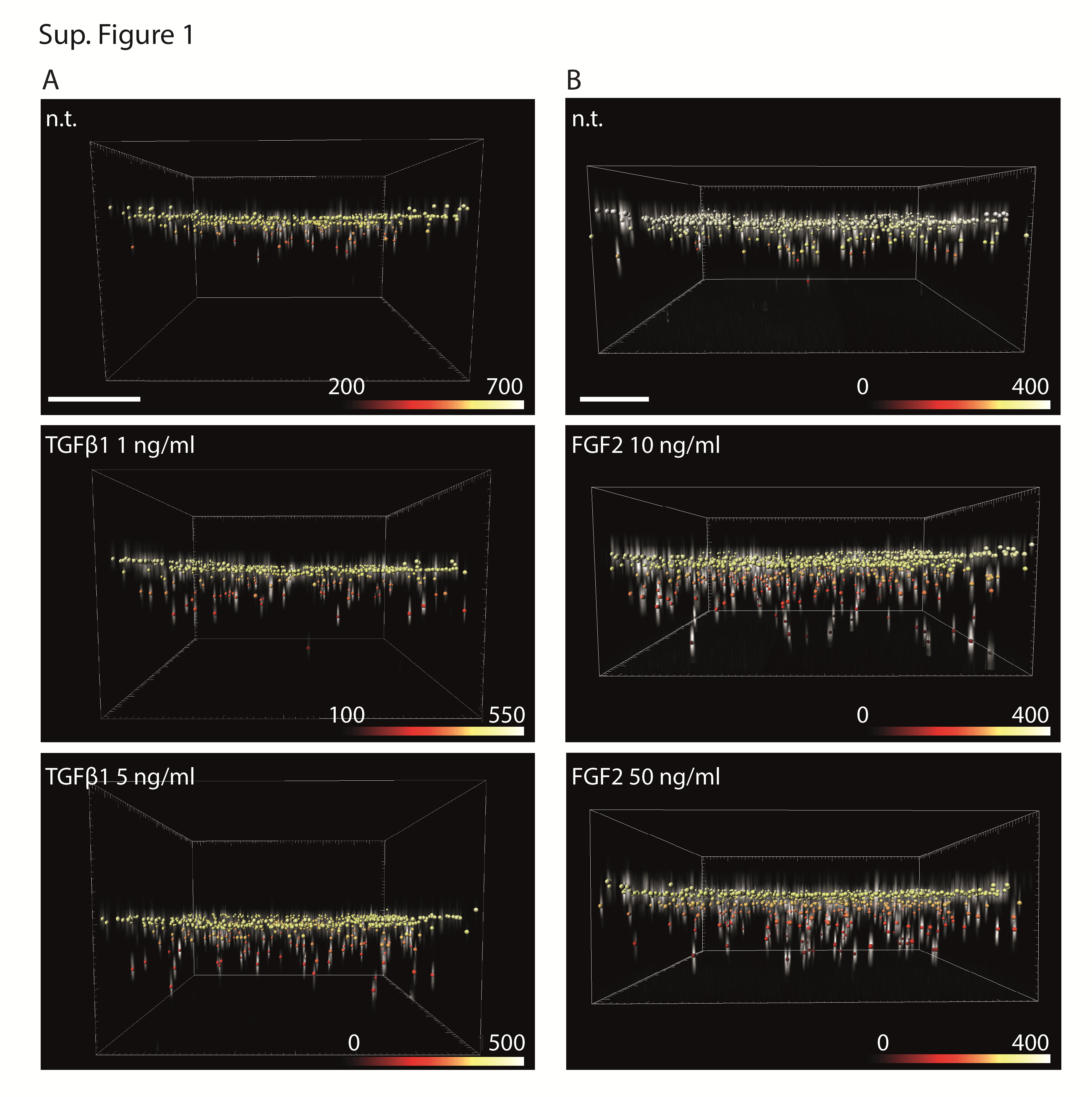
**


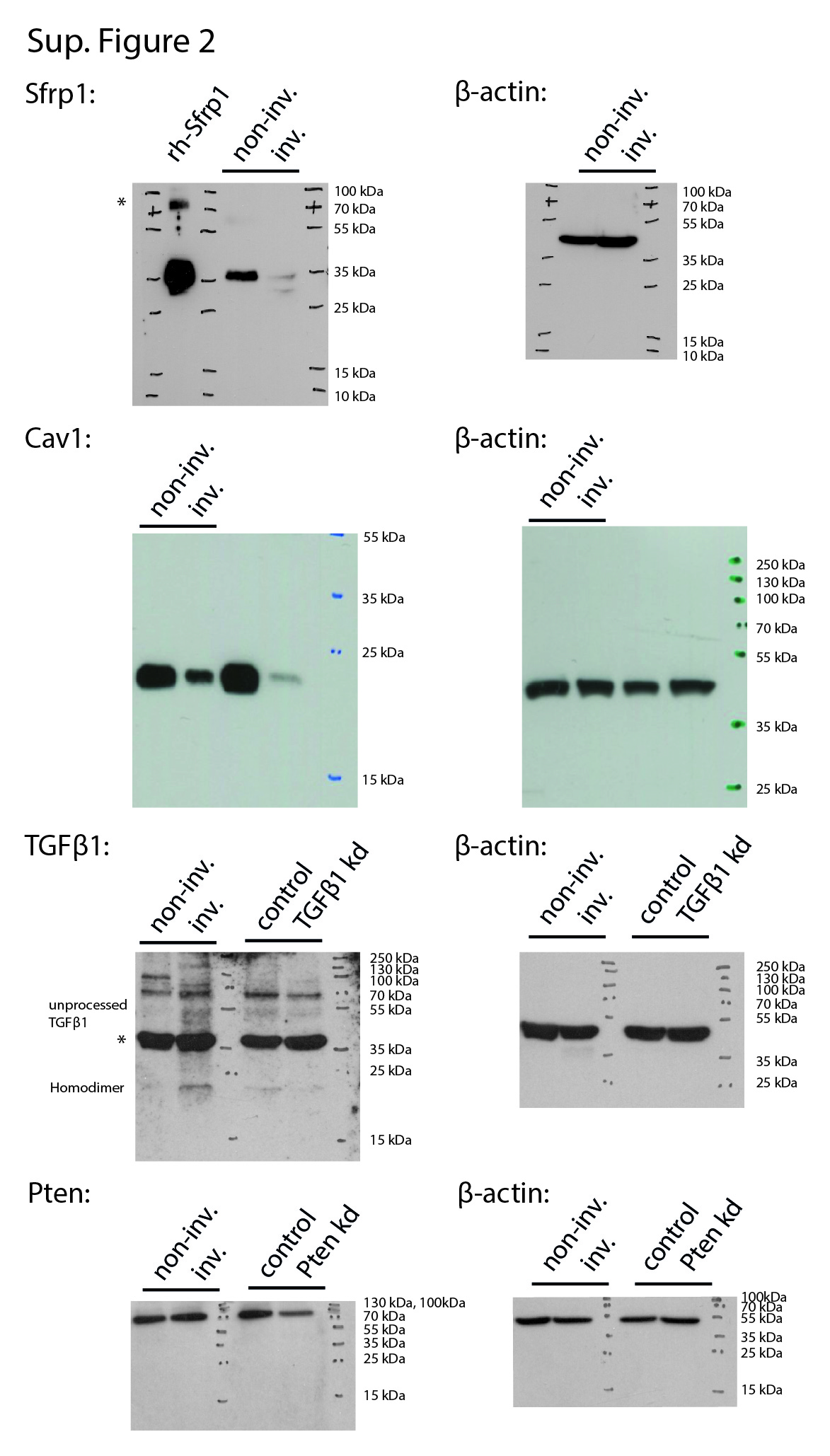

Supplement: Supplementary Information [file srep12673-s1.doc]
